# Post-COVID-19 condition after SARS-CoV-2 infection during pregnancy: a population-based questionnaire cohort study

**DOI:** 10.3389/fmed.2025.1674554

**Published:** 2026-01-21

**Authors:** Cecilie Bøge Paulsen, Cecilie Isgaard, Lone Krebs, Anna J. M. Aabakke

**Affiliations:** 1 Department of Anesthesiology, Copenhagen University Hospital – Hvidovre-Amager, Copenhagen, Denmark; 2 Department of Obstetrics and Gynecology, Copenhagen University Hospital – Holbæk, Holbæk, Denmark; 3 Department of Obstetrics and Gynecology, Copenhagen University Hospital – Hvidovre-Amager, Copenhagen, Denmark; 4 Department of Clinical Medicine, University of Copenhagen, Copenhagen, Denmark

**Keywords:** COVID-19, post-acute COVID-19 syndrome (MeSH), postpartum period (source: MeSH NLM), pregnancy, questionnaires (MeSH), surveys

## Abstract

**Introduction:**

Post-COVID-19 condition encompasses various symptoms that can occur after the acute SARS-CoV-2 infection. The risk of developing symptoms of the post-COVID-19 condition is higher in women infected during pregnancy than in non-obstetric cohorts.

**Method:**

We conducted a nationwide, prospective, population-based cohort study based on questionnaire data investigating the extent of post-COVID-19 condition after SARS-CoV-2 infection during pregnancy and whether the condition was related to the trimester at infection or the severity of infection. All women with a positive SARS-CoV-2 test during pregnancy in Denmark between 1 March 2020 and 17 November 2021 were invited to participate by responding to an online questionnaire.

**Result:**

Overall, 2,406 women met our inclusion criteria and were invited to participate by receiving an online questionnaire. In total, 1,007 (41.9%) responded to the survey, and 920 (38.2%) completed ≥70% of the items in the questionnaire and were included in the analysis. In total, 636 (69.1%) experienced a symptom of post-COVID-19 condition at any given time after SARS-CoV-2 infection, and 465 (50.1%) had at least one symptom of post-COVID-19 condition lasting ≥8 weeks after primary infection with SARS-CoV-2. The most common symptoms were fatigue, a change in the sense of smell and taste, and hair loss. The onset of most symptoms was within the first 4 weeks after infection with SARS-CoV-2. The median duration of symptoms differed substantially, ranging from 4 to 40 weeks, with forgetfulness lasting the longest. Participants who experienced symptoms during their primary SARS-CoV-2 infection were more likely to report symptoms of post-COVID-19 condition. However, we found no association between disease severity or trimester of infection and subsequent post-COVID-19 condition.

**Conclusion:**

Half of the women with SARS-CoV-2 infection during pregnancy had symptoms of post-COVID-19 condition lasting ≥8 weeks. The likelihood of reporting symptoms of post-COVID-19 condition increased when having symptomatic SARS-CoV-2 infection during pregnancy. Nonetheless, we found no association between severe disease requiring hospital admission or the trimester of infection and the subsequent development of post-COVID-19 condition.

## 1 Introduction

Post-COVID-19 condition has been defined by the World Health Organization (WHO) as a condition that “*occurs in individuals with a history of probable or confirmed SARS-CoV-2 infection, usually 3 months from the onset of COVID-19 with symptoms that last for at least 2 months and cannot be explained by an alternative diagnosis”* (1) meaning a condition that occurs approximately 3 months after a SARS-CoV-2 infection and that lasts more than 2 months without being explained by an alternative diagnosis. The total global prevalence of post-COVID-19 condition has been estimated to be 43% overall and 34% in exclusively non-hospitalized people in a systematic review and meta-analysis, with considerable variation in prevalence among the included studies (9–81%) (2).

Previous studies on the relationship between pregnancy and post-COVID-19 condition (3–7) have found that the symptoms of post-COVID-19 condition developed by women with SARS-CoV-2 infection in pregnancy are similar to the symptoms seen in non-obstetric cohorts (3–5). On the other hand, previous studies investigating post-COVID-19 condition among pregnant women are few (3–7), and none include a nationwide study population. However, one study found that women with even mild symptoms of SARS-CoV-2 infection during pregnancy had a greater risk of developing post-COVID-19 condition than did women in a non-pregnant control group (8). The prevalence of post-COVID-19 condition has been shown to correlate with the severity of the SARS-CoV-2 infection during pregnancy, with a higher prevalence among women with severe symptoms than in women with mild or no symptoms (3). Pregnant women infected with SARS-CoV-2 during the first and third trimesters (4, 9) and women who required hospitalization due to COVID-19 have a higher risk of developing post-COVID-19 condition (4).

The objective of this cohort study was to investigate the extent of post-COVID-19 condition after SARS-CoV-2 infection during pregnancy in a nationwide cohort. Moreover, we aimed to investigate specific symptoms characteristic of post-COVID-19 condition, and whether there was an association between the occurrence of post-COVID-19 condition and the trimester of infection or the severity of infection.

## 2 Methods

This was a nationwide, prospective, population-based cohort study based on questionnaire data. We included pregnant women with a positive SARS-CoV-2 test between 1 March 2020 and 17 November 2021 in Denmark who were registered in the Danish COVID-19 in Pregnancy Database (DCOD) (10–12). All women in Denmark with a positive SARS-CoV-2 test during pregnancy between 1 March 2020 and 31 May 2021 were registered in the DCOD, as previously described (11, 12). From 1 June 2021 DCOD included: (A) pregnant women with a severe pregnancy outcome after SARS-CoV-2 infection during pregnancy defined as intrauterine fetal death, delivery of a small for gestational age child ≤22% (−2SD), or preterm delivery before gestational age (GA) 37 weeks but within 28 days of infection and (B) pregnant women with severe infection defined as the requirement of oxygen supplementation or admission to an intensive care unit within 28 days of infection. The participants included in DCOD were followed up on until 6 weeks after delivery.

The symptom questionnaire was based on previously described symptoms of post-COVID-19 condition after SARS-CoV-2 infection in non-pregnant populations (13, 14). The symptoms included fatigue, sleep difficulties, change in the sense of smell and taste, difficulty concentrating, difficulty breathing, headache, pain with deep breaths, dizziness, heart palpitations, chest pains, hair loss, cough, forgetfulness, skin rash/itchy skin, joint and/or muscle pain, diarrhea, nausea and/or vomiting, depression, and anxiety attacks. The questionnaire also assessed women’s self-perceived cause of the symptoms (SARS-CoV-2 infection or pregnancy/maternity) (Supplementary File S1). The questionnaire was initially validated in women included in the DCOD from one obstetrics department (*n* = 33), and single questions were edited to clarify according to the results of the unpublished pilot study. The symptom questionnaire was created in EasyTrial^1^ and distributed through a personal digital mailbox (e-Boks) between 17 November and 23 December 2021. E-Boks is a secure platform for digital communication used by public authorities and enterprises in Denmark (15). Reminders were sent out twice to secure data completeness. Informed consent was obtained when the women answered the questionnaire. In accordance with the WHO definition, symptoms of post-COVID-19 condition were defined as symptoms arising after the primary SARS-CoV-2 infection and lasting at least 8 weeks (1). Demographic and clinical characteristics of the women were obtained from the DCOD. The date of the SARS-CoV-2 infection was defined as the date of the first positive SARS-CoV-2 test during pregnancy. Severe SARS-CoV-2 infection was defined as admission to the hospital due to SARS-CoV-2 symptoms, with admission and discharge on two different dates and a positive SARS-CoV-2 test within 14 days of admission.

Normally distributed data were presented as the mean with standard deviation. Non-normally distributed data were presented as median with interquartile range (IQR). The associations between post-COVID-19 condition and trimester at primary SARS-CoV-2 infection, hospital admission due to symptoms of COVID-19, and whether the women had symptoms during the primary infection were analyzed with the Pearson chi-square test. Data were analyzed using SPSS Statistics (IBM, version 29.0). The study was reported according to the Strengthening the Reporting of Observational Studies in Epidemiology guidelines (16, 17). The study was approved by the regional Data Protection Agency in Region Zealand (reg. no. REG-022-2020) and the Danish Patient Safety Authority (reg. no. 31-1521-252).

## 3 Results

**Figure 1:**
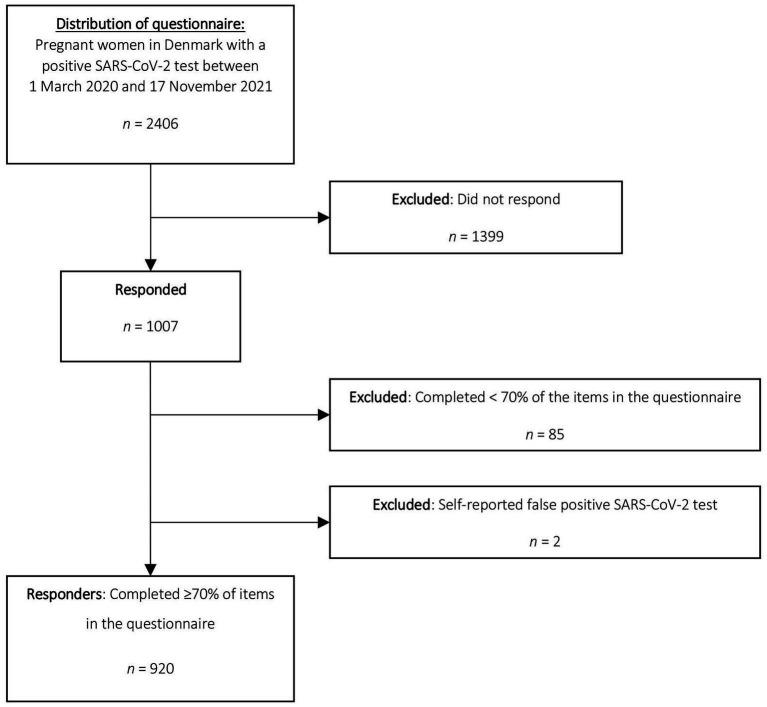
Flowchart of potential participants.

In total, 2,406 women with a positive SARS-CoV-2 test during pregnancy were invited to participate. Among these, 920 (38.2%) responded to the questionnaire and completed ≥70% of items in the questionnaire and were defined as responders and included in the analysis. The women who did not respond to the questionnaire, completed <70% of the items in the questionnaire, or reported having a false-positive SARS-CoV-2 test were defined as non-responders and excluded from the analysis (Figure 1).

Characteristics of responders (*n* = 920) and non-responders (*n* = 1,486) are shown in Table 1. Responders were less likely to smoke, were more often of Danish ethnicity, and more often delivered preterm before GA 37 weeks and experienced intrauterine fetal death. When receiving the questionnaire, 906 (99.1%) had delivered. The median duration between delivery and receiving the questionnaire was 211 days (IQR 144–286 days), and the median time from infection to response to the questionnaire was 341 days (IQR 297–374 days). SARS-CoV-2 vaccination was offered to pregnant women in Denmark from 21 July 2021, and 11 (1.2%) of the participants in this study were SARS-CoV-2 vaccinated. Of the responders, 773 (84.0, 95% CI 81.5–86.2) experienced symptoms of SARS-CoV-2 at the time of infection during pregnancy, and 636 (69.1, 95% CI 66.1–72.0) experienced a symptom of post-COVID-19 condition at any given time. Among responders, 461 (50.1, 95% CI 47.3–53.8) and 407 (44.2, 95% CI 41.1–47.5) had at least one symptom of post-COVID-19 condition lasting more than 8 and 12 weeks, respectively. Among the responders, 168 (18.4%) felt that their general health condition had deteriorated after their SARS-CoV-2 infection, 708 (77.4%) felt that it was unchanged, whereas 39 (4.2%) reported that it had improved. A total of 60 women (6.5, 95% CI 5.1–8.3) stated that they had consulted a physician due to a post-COVID-19 condition, and 21 (2.3, 95% CI 1.5–3.5) had received treatment for the condition. The majority of women in our study population would have had SARS-CoV-2 infection not requiring hospital contact and would thus not have received antivirals, as this was not routinely given to less severe infections. When examining the influence of the SARS-CoV-2 variant, we defined four calendar periods where different circulating variants were dominant: Index variant (1 March 2020–31 December 2020), Alpha variant (15 March–30 June 2021), Delta variant (15 July–15 November 2021), Omicron variant (28 December 2021 to end of follow-up), and Intermediate periods (between the defined variant periods) (18). Table 2 presents the prevalence, time of debut, and duration of symptoms reported by the responders and their self-perceived cause of the symptoms (SARS-CoV-2 infection or pregnancy/maternity). The most common symptoms at any given time after the SARS-CoV-2 infection were fatigue (38.6, 95% CI 35.5–41.8), change in the sense of smell (32.6, 95% CI 29.6–35.7) or taste (27.4, 95% CI 24.6–30.4), and hair loss (21.0, 95% CI 18.5–23.8). More than 90% of responders reported that they believed that the change in smell and/or taste was due to the SARS-CoV-2 infection. This was the case for only 43.1 and 8.9% of the responders when asked about fatigue and hair loss. The least common symptoms were anxiety attacks (4.6, 95% CI 3.4–6.2) and chest pain (4.6, 95% CI 3.4–6.1). When only considering symptoms lasting ≥8 weeks, the most common symptoms were the same, but with change in the sense of smell being the most common (21.7, 95% CI 19.1–24-5) followed by fatigue (19.1, 95% CI 16.7–21.8), change in the sense of taste (16.2, 95% CI 14.0–18.8), and hair loss (14.0, 95% CI 11.9–16.4). The duration of the symptoms differed substantially. Fatigue had a median duration of 8.0 weeks (IQR 4.0–20.0), whereas changes in sense of smell and taste, together with hair loss, had median durations between 14.0 and 18.0 weeks. The symptom with the longest duration was forgetfulness (40.0 weeks, IQR 12.0–52.0). Forgetfulness was reported by 17.7%, but considered related to COVID-19 by only 38.3% of them. The symptoms with the shortest durations were pain when breathing (4.0 weeks, IQR 2.0–20.0) and chest pain (4.5 weeks, IQR 2.0–30.0). Most symptoms appeared within the first 4 weeks after infection with SARS-CoV-2. Over one-third of the participants stated that they did not know whether a symptom was related to COVID-19 or to pregnancy/maternity in 11 out of the 19 symptoms investigated.

**Table 1:** Characteristics of responders* and non-responders to the questionnaire about post-COVID-19 condition among women with a positive SARS-CoV-2 test during pregnancy.

*n* (%)	Responders (Completeness ≥ 70%) (*n* = 920)	Non-responders (Completeness < 70%) (*n* = 1,484)
Maternal characteristics		
Age (years at positive test)		
<25	63 (6.8)	196 (13.2)
25–34	685 (74.5)	1,010 (68.2)
≥35	172 (18.7)	275 (18.6)
Mean (SD)	31.0 (4.5)	30.4 (5.0)
Missing	2	3
Ethnicity		
Foreign-born^a^	133 (17.2)	459 (39.6)
Missing	147	326
BMI		
<25	487 (59.4)	759 (60.0)
25–29.9	196 (23.9)	309 (24.4)
30–34.9	94 (11.5)	131 (10.3)
>35	43 (5.2)	67 (5.3)
Median (IQR)	24.1 (21.7–27.6)	23.8 (21.3–27.7)
Missing	102	218
Preexisting medical problems^b^		
Asthma	29 (3.5)	67 (5.2)
Missing	88	187
Other preexisting medical problems	127 (15.2)	188 (14.5)
Missing	88	191
Smoking during pregnancy	32 (4.0)	104 (8.3)
Missing	145	329
Pregnancy characteristics		
Parity		
Multiparous	432 (51.7)	753 (58.0)
Missing	87	185
Pregnancy outcome		
Termination of pregnancy before GA 22 weeks	12 (1.3)	45 (3.1)
Pregnancy loss before GA 22 weeks	20 (2.2)	33 (2.2)
Delivered > GA 22	883 (95.8)	1,392 (93.8)
Missing	7	14
COVID characteristics		
GA at infection^c^		
First trimester	131 (14.2)	227 (15.3)
Second trimester	494 (53.7)	719 (48.6)
Third trimester	292 (31.8)	533 (36.0)
Median (IQR)	22w4d (15w1d–30w1d)	23w3d (15w1d–31w5d)
Missing	3	5
Dominant virus variant at time of infection^d^		
Index/Wuhan	552 (60.1)	891 (60.2)
Intermediate period Index-Alpha	202 (22.0)	312 (21.1)
Alpha	153 (16.7)	276 (18.6)
Delta	>8	< 3
Other Intermediate periods (between Alpha-Delta and Delta-Omicron)	< 3	< 3
Missing	2	3
Admission status due to infection^e^		
Not admitted	818 (88.9)	1288 (86.9)
Admission due to reasons other than COVID-19 (including delivery)	74 (8.0)	139 (9.4)
Admission due to COVID symptoms	28 (3.0)	55 (3.7)
Missing	2	2
COVID-19 vaccinated^f^	11 (1.2)	18 (1.2)
Missing	384 (41.7)	736 (50.0)
Delivery characteristics		
Deliveries	*n* = 883	*n* = 1,392
GA at delivery	40w1d (39w1d–41w0d)	40w0d (39w1d–40w6d)
Preterm birth < GA 37	62 (7.0)	69 (5.0)
Multiparous	12 (1.4)	23 (1.7)
Mode of delivery		
Vaginal delivery	716 (81.1)	1,098 (78.9)
Emergency cesarean delivery	97 (11.0)	172 (12.4)
Elective cesarean delivery	70 (7.9)	121 (8.7)
Missing	0	1
Birth outcomes	*n* = 895	*n* = 1,415
IUFD/stillborn	10 (1.1)	< 3 (< 0.2)
Neonatal outcomes		
Live born children	*n* = 885	*n* > 1,412
NICU admission	77 (8.7)	154 (10.9)

Data are presented as count (%), median (SD), or median (interquartile range).

GA, gestational age; BMI, body mass index; IUFD, intrauterine fetal death.

a Foreign-born was defined as not born in Denmark.

b Preexisting medical problems included cardiac (congenital or acquired), renal, endocrine (for example, hypo- or hyperthyroidism), psychiatric (requiring medication), hematological (for example, sickle cell anemia or thrombophilia), inflammatory diseases (for example, inflammatory bowel disease), autoimmune diseases, previous gastric bypass operation, cancer, and HIV.

c Gestational age at infection was defined as GA at the date of the first positive PCR test or antibody test.

d Time periods of dominant virus variants in Denmark according to the Danish Covid-19 Genome Consortium available at https://www.covid19genomics.dk/statistics.

e Admission to the hospital was defined as admission and discharge on two different dates and a positive test within 14 days before or during admission.

f At least one COVID-19 vaccination prior to infection.

*A participant was defined as a responder when completing ≥70% of items in the questionnaire. The women invited to participate who did not respond to the questionnaire, completed <70% of the items in the questionnaire, or reported having a false positive test were defined as non-responders.

**Table 2:** Post-COVID-19 symptoms reported by responders infected with *SARS-CoV-2* during pregnancy, including prevalence and duration of the symptoms, and the responders’ self-perceived cause of the symptoms.

Symptoms of post-COVID-19 condition	Total responders of specific question, *n*	Yes (%)/95% CI	Weeks until debut of symptoms, median (IQR)	Duration of symptom in weeks, median (IQR)	Responders with duration of a specific symptom ≥8 weeks, *n* (%)/95% CI	The responders self-perceived cause of the symptoms, *n* (%)			
						Post-COVID-19 condition	Pregnancy and/or maternity	Do not know	Missing
Fatigue	920	355 (38.6)/0.35–0.42	0.5 (0.0–2.8)	8.0 (4.0–20.0)	176 (19.1)/0.17–0.22	153 (43.1)	78 (22.0)	115 (32.4)	9 (2.5)
Sleeping difficulties	918	128 (13.9)/0.12–0.16	2.0 (0.0–8.0)	16.0 (5.0–38.0)	74 (8.1)/0.06–0.10	33 (25.8)	44 (34.4)	49 (38.3)	2 (1.6)
Change in sense of smell	918	299 (32.6)/0.30–0.36	0.0 (0.0–2.0)	18.0 (6.0–46.0)	199 (21.7)/0.19–0.24	272 (91.0)	4 (1.3)	15 (5.0)	8 (2.7)
Change in sense of taste	912	250 (27.4)/0.25–0.30	0.0 (0.0–1.0)	14.0 (4.0–47.0)	148 (16.2)/0.14–0.19	225 (90.0)	5 (2.0)	14 (5.6)	6 (2.4)
Difficulty concentrating	918	152 (16.6)/0.14–0.19	1.0 (0.0–4.0)	24.0 (8.0–52.0)	95 (10.3)/0.09–0.12	84 (55.3)	13 (8.6)	50 (32.9)	5 (3.3)
Difficulty breathing	919	131 (14.3)/0.12–0.17	0.0 (0.0–2.0)	10.0 (4.0–33.0)	79 (8.6)/0.07–0.11	103 (78.6)	4 (3.1)	19 (14.5)	5 (3.8)
Headache	920	166 (18.0)/0.16–0.21	1.0 (0.0–3.0)	10.5 (2.9–40.0)	79 (8.6)/ 0.07–0.11	97 (58.4)	10 (6.0)	52 (31.3)	7 (4.2)
Pain when breathing deeply	917	49 (5.3)/0.04–0.07	1.0 (0.0–2.8)	4.0 (2.0–20.0)	17 (1.9)/0.01–0.03	37 (75.5)	0 (0.0)	10 (20.4)	2 (4.1)
Dizziness	918	119 (13.0)/0.11–0.15	1.0 (0.0–10.0)	11.0 (4.0–39.0)	63 (6.9)/0.05–0.09	52 (43.7)	16 (13.4)	49 (41.2)	2 (1.7)
Heart palpitations	918	67 (7.3)/0.06–0.09	3.0 (0.0–8.0)	11.0 (4.3–34.8)	33 (3.6)/0.03–0.05	28 (41.8)	11 (16.4)	26 (38.8)	2 (3.0)
Chest pain	916	42 (4.6)/0.03–0.06	4.0 (0.8–20.5)	4.5 (2.0–30.0)	15 (1.6)/0.01–0.03	17 (40.5)	5 (11.9)	19 (45.2)	1 (2.4)
Hair loss	914	192 (21.0)/0.18–0.24	17.5 (5.75–28.5)	15.0 (8.0–32.5)	128 (14.0)/0.12–0.16	17 (8.9)	115 (59.9)	59 (30.7)	1 (0.5)
Cough	917	85 (9.3)/0.08–0.11	1.0 (0.0–4.0)	10.0 (4.0–30.0)	45 (4.9)/0.04–0.07	65 (76.5)	1 (1.2)	13 (15.3)	6 (7.1)
Forgetfulness	915	162 (17.7)/0.15–0.20	2.0 (0.0–8.5)	40.0 (12.0–52.0)	108 (11.8)/0.10–0.14	62 (38.3)	33 (20.4)	61 (37.7)	6 (3.7)
Skin rash/itchy skin	915	61 (6.7)/0.05–0.08	4.0 (1.0–28.0)	10.0 (4.0–39.3)	30 (3.3)/0.02–0.05	20 (32.8)	9 (14.8)	30 (49.2)	2 (3.3)
Joint and/or muscle pain	914	129 (14.1)/0.12–0.17	2.0 (0.0–10.0)	20.0 (4.8–48.0)	77 (8.4)/0.07–0.10	54 (41.9)	18 (14.0)	52 (40.3)	5 (3.9)
Diarrhea, nausea, and/or vomiting	910	55 (6.0)/0.05–0.08	2.0 (0.0–14.8)	6.0 (2.0–28.5)	21 (2.3)/0.02–0.04	15 (27.3)	13 (23.6)	25 (45.5)	2 (3.6)
Depression	914	95 (10.4) /0.09–0.13	1.5 (0.0–7.0)	20.0 (8.0–44.3)	55 (6.0)/0.05–0.08	33 (34.7)	23 (24.2)	33 (34.7)	6 (6.3)
Anxiety attacks	912	42 (4.6)/0.03–0.06	10.0 (1.0–29.5)	–	–	11 (26.2)	10 (23.8)	20 (47.6)	1 (2.4)

CI, confidence interval; IQR, interquartile range.

We found no association between trimester of primary infection with SARS-CoV-2 (*p* = 0.624) or severe infection with SARS-CoV-2 requiring admission to hospital (OR 1.34, 95% CI 0.57–3.26) and the later reporting of post-COVID-19 condition. Women with symptoms during primary infection with SARS-CoV-2 were more likely to report symptoms of post-COVID-19 condition (OR 4.88, 95% CI 3.37–7.07) (Supplementary File S2).

## 4 Discussion

Among 920 responders to a questionnaire about post-COVID-19 condition following SARS-CoV-2 infection during pregnancy, 636 (69.1%) reported symptoms of post-COVID-19 condition, and 465 (50.1%) had symptoms lasting ≥8 weeks. The most common symptoms were fatigue, a change in sense of smell and taste, and hair loss. Participants who experienced symptoms during their primary SARS-CoV-2 infection were more likely to report symptoms of post-COVID-19 condition. However, we found no association between severe primary infection with SARS-CoV-2 requiring hospital admission or trimester at infection and the later development of post-COVID-19 condition.

This study found that 69.1% of the responders reported symptoms of post-COVID-19 condition, but only 50.1% fulfilled the WHO criteria for post-COVID-19 condition and reported symptoms lasting ≥8 weeks. Previous studies have found prevalences of post-COVID-19 condition in obstetric cohorts ranging from 25 to 75.9% (4, 7, 8). The inconsistency is probably related to the definition of post-COVID-19 condition. The study with the highest reported prevalence had a median duration of symptoms of 8.5 weeks, and the study would probably have found a lower prevalence if the WHO definition had been used (8). However, the study reporting the lowest prevalence reported only symptoms lasting ≥8 weeks (7). Our finding that fatigue, changes in the sense of smell and taste, and hair loss were the most common symptoms align with the findings of other studies investigating post-COVID-19 condition in obstetric cohorts (3, 4, 8). According to the WHO definition, post-COVID-19 condition usually appears 3 months after SARS-CoV-2 infection (1). In our study, only hair loss appeared more than 3 months after the infection with SARS-CoV-2 [17.5 weeks (IQR 5.75–28.5)]. However, the most common symptoms among our participants were seen earlier than 3 months after infection but lasted ≥8 weeks and can thus be considered as symptoms of post-COVID-19 condition. Additionally, the timing of symptoms in our study might be affected by recall bias because the median time between SARS-CoV-2 infection and response to the questionnaire was almost a year. The symptoms, including pain when breathing deeply, chest pain, diarrhea, nausea, and/or vomiting, all had a median duration of <8 weeks and would, therefore, not be considered as a post-COVID-19 condition according to the WHO definition. Nevertheless, these symptoms are related to a previous SARS-CoV-2 infection and might very well be short-term sequelae of the infection. Two-thirds of the participants assumed that pain when breathing deeply was associated with COVID-19, whereas a smaller number thought this was the case with the other symptoms lasting <8 weeks. Overall, one-third of participants were in doubt whether most symptoms were related to COVID-19 or to maternity/pregnancy, illustrating the similarity between post-COVID-19 condition and general maternity complaints. We found no association between the trimester of primary infection with SARS-CoV-2 and the occurrence of post-COVID-19 condition, in contrast to a previous study that reported higher risks if infection occurred during the first and third trimesters (4). The population studied might explain this discrepancy, because the Danish population was universally tested during the pandemic, and our study population is dominated by pregnant women with mild symptoms and no hospital contact. Additionally, we found that participants with symptoms during infection with COVID-19 were more likely to report symptoms of post-COVID-19 condition, correlating with previous studies conducted in obstetric cohorts (3, 4). A former study showed that having symptoms such as cough and myalgia/arthralgia during primary infection increased the risk of post-COVID-19 condition (3). This poses the theory that specific symptoms during primary infection increase the risk of later post-COVID-19 condition. Several studies have found that having moderate to severe symptoms, including hospitalization during primary SARS-CoV-2 infection, leads to a higher risk of post-COVID-19 condition in both obstetric and non-obstetric cohorts (3, 4, 6, 13, 19, 20). However, we found no such association. This might be explained by the few severe cases in our cohort; hence, our study may be underpowered to investigate these associations.

The major strengths of this study are the large study population and the study design, because all pregnant women with a positive SARS-CoV-2 test in Denmark were invited to participate. Additionally, women were asked about symptoms up to 1-year post-infection. The study also has limitations, the primary being the absence of a control group. This limits the generalizability of our findings regarding the occurrence of post-COVID-19 condition within the obstetric population, as some of the symptoms registered might also have been related to the pregnancy. However, due to the relatively recent definition of post-COVID-19 condition (1), defining it as symptoms lasting ≥8 weeks, this study contributes to the knowledge about and occurrence of post-COVID-19 condition among an obstetric population. This study was based on a symptom questionnaire. Participants experiencing mild or no symptoms might have been less likely to respond to the questionnaire or complete ≥70% of the items in the questionnaire, causing an overestimation of post-COVID-19 condition in our study. The long interval between infection and the distribution of the questionnaire, combined with social factors related to the postpartum period, might have further influenced the response rates, introducing a selection bias in our study. A few participants were infected during the delta-period. However, relatively more women among responders were infected during the delta period. Additionally, the birth outcomes (Table 1) show that 10 (1.1%) of the responders experienced intrauterine fetal death or stillbirth, compared to less than 3 (<0.2%) of the non-responders. The Delta variant has been shown to cause more severe outcomes in pregnancy (21). Women with traumatic outcomes might have been more inclined to respond, potentially introducing bias in the study—although the relative proportion of traumatic outcomes was very low. The median time from infection to response to the questionnaire was 341 days (IQR 297–374 days), increasing the likelihood of recall bias, which might have influenced the results and estimation of duration of symptoms. Non-responders were statistically more likely to be foreign-born, to smoke during pregnancy, and to terminate the pregnancy before GA 22 weeks. The language abilities of the women invited to participate were not known. Therefore, there is a possibility that women not mastering the Danish language were invited to respond to a questionnaire in Danish. This might have led to non-response bias. Smoking during and termination of pregnancy may be considered sensitive topics, and the invited participants might, therefore, potentially be reluctant to respond, leading to non-response bias (22).

In conclusion, more than half of women with SARS-CoV-2 infection during pregnancy had symptoms of post-COVID-19 condition lasting ≥8 weeks. The most common symptoms were fatigue, a change in the sense of smell and taste, and hair loss. Participants who experienced symptoms during the SARS-CoV-2 infection were more likely to report symptoms of post-COVID-19 condition. Indicating that health professionals in contact with postpartum women must have increased focus on post-COVID-19 condition if infected during pregnancy. Nonetheless, we found no association between severe disease requiring hospital admission or the trimester of infection and the subsequent development of post-COVID-19 condition.

## Data Availability

The raw data supporting the conclusions of this article will be made available by the authors, without undue reservation.

## Footnotes

1
easytrial.net
