# Supplementary material for: Post-COVID-19 condition after SARS-CoV-2 infection during pregnancy: a population-based questionnaire cohort study
Source: Front Med (Lausanne). 2026 Jan 21;12:1674554. doi: 10.3389/fmed.2025.1674554 (PMC12868251; doi:10.3389/fmed.2025.1674554)
Supplement: Supplementary file 1 [file Table_1.docx]

**Supplementary file S1 - Symptom questionnaire**

1. Was your COVID-19 test during pregnancy a false positive?

Answer options: Yes or no

If the option “yes” was selected, participants were presented with a text box where they could elaborate on how it was confirmed that the COVID-19 test was a false positive.

1. Have you had symptoms of COVID-19 such as fever, muscle aches, influenza symptoms, or decreased sense of smell and taste?

Answer options: Yes or no

1. Are you still pregnant?

Answer options: Yes or no

1. How many days did you have COVID-19 symptoms such as fever, muscle aches, influenza symptoms, and decreased sense of smell and taste?

Answer options: Numerical field in which participants enter values from 1 to 600 days.

1. In the period after you had COVID-19, did you experience **fatigue** that you did not experience before COVID-19?

Answer options: Yes or no

| If yes: |  |  |
| --- | --- | --- |
|  | 5.1 | How many weeks went by after you had COVID-19 before you experienced **fatigue**?  Answer options: Numerical field in which participants enter values from 0 to 150 weeks. |
|  | 5.2 | Duration of **fatigue** in weeks  Answer options: Numerical field in which participants enter values from 0.5 to 150 weeks. |
|  | 5.3 | Severity of **fatigue** from 0 to 10, where 0 is no change in level of tiredness and 10 is exhaustion.  Answer options: VAS scale from 0 to 10; it is only possible to select whole values. |
|  | 5.4 | Do you think this symptom was due to pregnancy and/or period of maternity leave, or COVID-19?  Answer options:  - COVID-19  - Pregnancy and/or maternity  - Do not know |
|  | 5.5 | Do you still have this symptom?  Answer options: Yes or no |

1. In the period after you had COVID-19, have you had **difficulty sleeping**?

Answer options: Yes or no

| If yes: |  |  |
| --- | --- | --- |
|  | 6.1 | How many weeks went by after you had COVID-19 before you experienced **difficulty sleeping**?  Answer options: Numerical field in which participants enter values from 0 to 150 weeks. |
|  | 6.2 | Duration of **sleep difficulties** in weeks  Answer options: Numerical field in which participants enter values from 0.5 to 150 weeks. |
|  | 6.3 | The type of **sleep difficulties:**  Answer options:  - Difficulty falling asleep  - Many awakenings during the night  - Very tired  - Other  It was possible to choose several options. If the option “other” was selected, then a text box appeared where participants could comment on their other types of sleep problems. |
|  | 6.4 | Do you think this symptom was due to pregnancy and/or period of maternity leave, or COVID-19?  Answer options:  - COVID-19  - Pregnancy and/or maternity  - Do not know |
|  | 6.5 | Do you still have this symptom?  Answer options: Yes or no |

1. Has your **sense of smell** changed from before you had COVID-19 to after you had COVID-19?

Answer options: Yes or no

| If yes: |  |  |
| --- | --- | --- |
|  | 7.1 | Is your **sense of smell** better or worse?  Answer options: Better or worse |
|  | 7.2 | How many weeks went by after you had COVID-19 before you experienced a **change in sense of smell**?  Answer options: Numerical field in which participants enter values from 0 to 150 weeks. |
|  | 7.3 | Duration of **change in sense of smell** in weeks  Answer options: Numerical field in which participants enter values from 0.5 to 150 weeks. |
|  | 7.4 | Do you think this symptom was due to pregnancy and/or period of maternity leave, or COVID-19?  Answer options:  - COVID-19  - Pregnancy and/or maternity  - Do not know |
|  | 7.5 | Do you still have this symptom?  Answer options: Yes or no |

1. Has your **sense of taste** changed from before you had COVID-19 to after you had COVID-19?

Answer options: Yes or no

| If yes: |  |  |
| --- | --- | --- |
|  | 8.1 | Is your **sense of taste** better or worse?  Answer options: Better or worse. |
|  | 8.2 | How many weeks went by after you had COVID-19 before you experienced a **change in sense of taste**?  Answer options: Numerical field in which participants enter values from 0 to 150 weeks. |
|  | 8.3 | Duration of **change in sense of taste** in weeks  Answer options: Numerical field in which participants enter values from 0.5 to 150 weeks. |
|  | 8.4 | Do you think this symptom was due to pregnancy and/or period of maternity leave, or COVID-19?  Answer options:  - COVID-19  - Pregnancy and/or maternity  - Do not know |
|  | 8.5 | Do you still have this symptom?  Answer options: Yes or no |

1. Have you had more **difficulty concentrating** after you had COVID-19 compared to before you had COVID-19?

Answer options: Yes or no

| If yes: |  |  |
| --- | --- | --- |
|  | 9.1 | How many weeks went by after had COVID-19 before you experienced **difficulty concentrating**?  Answer options: Numerical field in which participants enter values from 0 to 150 weeks. |
|  | 9.2 | Duration of **difficulty concentrating** in weeks  Answer options: Numerical field in which participants enter values from 0.5 to 150 weeks. |
|  | 9.3 | Severity of **difficulty concentrating** rated from 0 to 10, where 0 is no difficulty concentrating and 10 is severe difficulty concentrating.  Answer options: VAS scale from 0 to 10; it is only possible to select whole values. |
|  | 9.4 | Do you think this symptom was due to pregnancy and/or period of maternity leave, or COVID-19?  Answer options:  - COVID-19  - Pregnancy and/or maternity  - Do not know |
|  | 9.5 | Do you still have this symptom?  Answer options: Yes or no |

1. After having COVID-19, have you had more **shortness of breath/difficulty breathing** compared to before you had COVID-19?

Answer options: Yes or no

| If yes: |  |  |
| --- | --- | --- |
|  | 10.1 | How many weeks went by after you had COVID-19 before you experienced **shortness of breath/difficulty breathing**?  Answer options: Numerical field in which participants enter values from 0 to 150 weeks. |
|  | 10.2 | Duration of **shortness of breath/difficulty breathing** in weeks  Answer options: Numerical field in which participants enter values from 0.5 to 150 weeks. |
|  | 10.3 | Severity of **shortness of breath/difficulty breathing** rated from 0 to 10, where 0 is no shortness of breath /difficulty breathing and 10 is shortness of breath /difficulty breathing at the slightest physical activity.  Answer options: VAS scale from 0 to 10; it is only possible to select whole values. |
|  | 10.4 | Do you think this symptom was due to pregnancy and/or period of maternity leave, or COVID-19?  Answer options:  - COVID-19  - Pregnancy and/or maternity  - Do not know |
|  | 10.5 | Do you still have this symptom?  Answer options: Yes or no |

1. Have you had more **headaches** after you had COVID-19 compared to before you had COVID-19?

Answer options: Yes or no

| If yes: |  |  |
| --- | --- | --- |
|  | 11.1 | How many weeks went by after you had COVID-19 before you experienced **headache**?  Answer options: Numerical field in which participants enter values from 0 to 150 weeks. |
|  | 11.2 | Duration of **headache** in weeks  Answer options: Numerical field in which participants enter values from 0.5 to 150 weeks |
|  | 11.3 | Severity of **headache** rated from 0 to 10, where 0 is no headache and 10 is the worst pain imaginable.  Answer options: VAS scale from 0 to 10; it is only possible to select whole values. |
|  | 11.4 | Do you think this symptom was due to pregnancy and/or period of maternity leave, or COVID-19?  Answer options:  - COVID-19  - Pregnancy and/or maternity  - Do not know |
|  | 11.5 | Do you still have this symptom?  Answer options: Yes or no |

1. Have you had **pain when you take deep breaths** after you had COVID-19?

Answer options: Yes or no

| If yes: |  |  |
| --- | --- | --- |
|  | 12.1 | How many weeks went by after you had COVID-19 before you experienced **pain when you take deep breaths**?  Answer options: Numerical field in which participants enter values from 0 to 150 weeks. |
|  | 12.2 | Duration of **pain when you take deep breaths** in weeks  Answer options: Numerical field in which participants enter values from 0.5 to 150 weeks. |
|  | 12.3 | Severity of **pain when you take deep breaths** rated from 0 to 10, where 0 is no pain and 10 is the worst pain imaginable.  Answer options: VAS scale from 0 to 10; it is only possible to select whole values. |
|  | 12.4 | Do you think this symptom was due to pregnancy and/or period of maternity leave, or COVID-19?  Answer options:  - COVID-19  - Pregnancy and/or maternity  - Do not know |
|  | 12.5 | Do you still have this symptom?  Answer options: Yes or no |

1. Have you felt more **dizzy** after you had COVID-19?

Answer options: Yes or no

| If yes: |  |  |
| --- | --- | --- |
|  | 13.1 | How many weeks went by after you had COVID-19 before you experienced **dizziness**?  Answer options: Numerical field in which participants enter values from 0 to 150 weeks. |
|  | 13.2 | Duration of **dizziness** in weeks  Answer options: Numerical field in which participants enter values from 0.5 to 150 weeks. |
|  | 13.3 | Severity of **dizziness** rated from 0 to 10, where 0 is no dizziness and 10 is dizziness several times a day.  Answer options: VAS scale from 0 to 10; it is only possible to select whole values. |
|  | 13.4 | Do you think this symptom was due to pregnancy and/or period of maternity leave, or COVID-19 ?  Answer options:  - COVID-19  - Pregnancy and/or maternity  - Do not know |
|  | 13.5 | Do you still have this symptom?  Answer options: Yes or no |

1. Do you have more **heart palpitations** after you have had COVID-19 compared to before had COVID-19?

Answer options: Yes or no

| If yes: |  |  |
| --- | --- | --- |
|  | 14.1 | How many weeks went by after you had COVID-19 before you experienced **heart palpitations**?  Answer options: Numerical field in which participants enter values from 0 to 150 weeks |
|  | 14.2 | Duration of **heart palpitations** in weeks  Answer options: Numerical field in which participants enter values from 0.5 to 150 weeks |
|  | 14.3 | Severity of **heart palpitations** rated from 0 to 10, where 0 is no heart palpitations and 10 is heart palpitations several times a day.  Answer options: VAS scale from 0 to 10; it is only possible to select whole values. |
|  | 14.4 | Do you think this symptom was due to pregnancy and/or period of maternity leave, or COVID-19?  Answer options:  - COVID-19  - Pregnancy and/or maternity  - Do not know |
|  | 14.5 | Do you still have this symptom?  Answer options: Yes or no |

1. Have you had more episodes of **chest pains** after you had COVID-19 than before you had COVID-19?

Answer options: Yes or no.

| If yes: |  |  |
| --- | --- | --- |
|  | 15.1 | How many weeks went by after you had COVID-19 before you experienced **chest pains**?  Answer options: Numerical field in which participants enter values from 0 to 150 weeks. |
|  | 15.2 | Duration of **chest pains** in weeks  Answer options: Numerical field in which participants enter values from 0.5 to 150 weeks. |
|  | 15.3 | Severity of **chest pains** rated from 0 to 10, where 0 is no pain and 10 is the worst pain imaginable.  Answer options: VAS scale from 0 to 10; it is only possible to select whole values. |
|  | 15.4 | Do you think this symptom was due to pregnancy and/or period of maternity leave, or COVID-19?  Answer options:  - COVID-19  - Pregnancy and/or maternity  - Do not know |
|  | 15.5 | Do you still have this symptom?  Answer options: Yes or no |

1. Do you suffer more from **hair loss** after you have had COVID-19 compared to before you had COVID-19?

Answer options: Yes or no

| If yes: |  |  |
| --- | --- | --- |
|  | 16.1 | How many weeks went by after you had COVID-19 before you experienced **hair loss**?  Answer options: Numerical field in which participants enter values from 0 to 150 weeks. |
|  | 16.2 | Duration of **hair loss** in weeks  Answer options: Numerical fields in which participants enter values from 0.5 to 150 weeks. |
|  | 16.3 | Do you think this symptom was due to pregnancy and/or period of maternity leave, or COVID-19 ?  Answer options:  - COVID-19  - Pregnancy and/or maternity  - Do not know |
|  | 16.4 | Do you still have this symptom?  Answer options: Yes or no |

1. Do you **cough** more often after you have had COVID-19 than before you had COVID-19?

Answer options: Yes or no

| If yes: |  |  |
| --- | --- | --- |
|  | 17.1 | How many weeks went by after you had COVID-19 before you experienced **coughing**?  Answer options: Numerical field in which participants enter values from 0 to 150 weeks. |
|  | 17.2 | Duration of **coughing** in weeks  Answer options: Numerical field in which participants enter values from 0.5 to 150 weeks. |
|  | 17.3 | Do you think this symptom was due to pregnancy and/or period of maternity leave, or COVID-19?  Answer options:  - COVID-19  - Pregnancy and/or maternity  - Do not know |
|  | 17.4 | Do you still have this symptom?  Answer options: Yes or no |

1. Are you more **forgetful** after you have had COVID-19 compared to before your COVID-19?

Answer options: Yes or no

| If yes: |  |  |
| --- | --- | --- |
|  | 18.1 | How many weeks went by after you had COVID-19 before you experienced **forgetfulness**?  Answer options: Numerical field in which participants enter values from 0 to 150 weeks. |
|  | 18.2 | Duration of **forgetfulness** in weeks  Answer options: Numerical field in which participants enter values from 0.5 to 150 weeks |
|  | 18.3 | Severity of **forgetfulness rated** from 0 to 10, where 0 is no forgetfulness and 10 is severe forgetfulness.  Answer options: VAS scale from 0 to 10; it is only possible to select whole values. |
|  | 18.4 | Do you think this symptom was due to pregnancy and/or period of maternity leave, or COVID-19?  Answer options:  - COVID-19  - Pregnancy and/or maternity  - Do not know |
|  | 18.5 | Do you still have this symptom?  Answer options: Yes or no |

1. Did you get a **skin rash** and/or have **itchy skin** after you had COVID-19?

Answer options: Yes or no

| If yes: |  |  |
| --- | --- | --- |
|  | 19.1 | How many weeks went by after you had COVID-19 before you experienced **skin rash** and/or had **itchy skin**?  Answer options: Numerical field in which participants enter values from 0 to 150 weeks |
|  | 19.2 | Duration of **skin rash** and/or **itchy skin** in weeks  Answer options: Numerical field in which participants enter values from 0.5 to 150 weeks |
|  | 19.3 | Severity of **skin rash** and/or **itchy skin** rated from 0 to 10, where 0 is no skin rash and/or itchy skin and 10 is a severe skin rash and/or very itchy skin.  Answer options: VAS scale from 0 to 10; it is only possible to select whole values. |
|  | 19.4 | Do you think this symptom was due to pregnancy and/or period of maternity leave, or COVID-19?  Answer options:  - COVID-19  - Pregnancy and/or maternity  - Do not know |
|  | 19.5 | Do you still have this symptom?  Answer options: Yes or no |

1. Do you have more/new **joint pain** and/or **muscle pain** after you have had COVID-19 compared to before your COVID-19?

Answer options: Yes or no

| If yes: |  |  |
| --- | --- | --- |
|  | 20.1 | How many weeks went by after you had COVID-19 before you experienced **joint pain** and/or **muscle pain**?  Answer options: Numerical field in which participants enter values from 0 to 150 weeks. |
|  | 20.2 | Duration of **joint pain** and/or **muscle pain** in weeks  Answer options: Numerical field in which participants enter values from 0.5 to 150 weeks. |
|  | 20.3 | Severity of **joint pain** and/or **muscle pain** rated from 0 to 10, where 0 is no pain and 10 is the worst pain imaginable.  Answer options: VAS scale from 0 to 10; it is only possible to select whole values. |
|  | 20.4 | Do you think this symptom was due to pregnancy and/or period of maternity leave, or COVID-19 infection?  Answer options:  - COVID-19  - Pregnancy and/or maternity  - Do not know |
|  | 20.5 | Do you still have this symptom?  Answer options: Yes or no |

1. Do you have more/worse **diarrhea,** **nausea,** and/or **vomiting** after you have had COVID-19 compared to before you had COVID-19?

Answer options: Yes or no

| If yes: |  |  |
| --- | --- | --- |
|  | 21.1 | How many weeks went by after you had COVID-19 before you experienced **diarrhea, nausea,** and/or **vomiting**?  Answer options: Numerical field in which participants enter values from 0 to 150 weeks. |
|  | 21.2 | Duration of **diarrhea, nausea,** and/or **vomiting** in weeks  Answer options: Numerical field in which participants enter values from 0.5 to 150 weeks. |
|  | 21.3 | Severity of **diarrhea, nausea,** and/or **vomiting** rated from 0 to 10, where 0 is no diarrhea, nausea, and/or vomiting and 10 is diarrhea, nausea, and/or vomiting daily.  Answer options: VAS scale from 0 to 10, it is only possible to select whole values. |
|  | 21.4 | Do you think this symptom was due to pregnancy and/or period of maternity leave, or COVID-19?  Answer options:  - COVID-19  - Pregnancy and/or maternity  - Do not know |
|  | 21.5 | Do you still have this symptom?  Answer options: Yes or no |

1. Have you been feeling **more depressed/down** after you had COVID-19 compared to before you had COVID-19?

Answer options: Yes or no

| If yes: |  |  |
| --- | --- | --- |
|  | 22.1 | How many weeks went by after you had COVID-19 before you experienced feeling **depressed/down**?  Answer options: Numerical field in which participants enter values from 0 to 150 weeks. |
|  | 22.2 | Duration of **depression/feeling down** in weeks  Answer options: Numerical field in which participants enter values from 0.5 to 150 weeks. |
|  | 22.3 | Severity of **depression/feeling down** rated from 0 to 10, where 0 is no depression and 10 is very depressed.  Answer options: VAS scale from 0 to 10; it is only possible to select whole values. |
|  | 22.4 | Do you think this symptom was due to pregnancy and/or period of maternity leave, or COVID-19?  Answer options:  - COVID-19  - Pregnancy and/or maternity  - Do not know |
|  | 22.5 | Do you still have this symptom?  Answer options: Yes or no |

1. In the period after you had COVID-19, have you had **anxiety attacks** that you had not experienced before?

Answer options: Yes or no

| If yes: |  |  |
| --- | --- | --- |
|  | 23.1 | How many weeks went by after you had COVID-19 before you experienced an **anxiety attack**?  Answer options: Numerical field in which participants enter values from 0 to 150 weeks. |
|  | 23.2 | How many new **anxiety attacks** have you experienced after you had COVID-19?  Numerical field in which participants enter values from 1 to 500 anxiety attacks. |
|  | 23.3 | Do you think this symptom was due to pregnancy and/or period of maternity leave, or COVID-19?  Answer options:  - COVID-19  - Pregnancy and/or maternity  - Do not know |
|  | 23.4 | Do you still have this symptom?  Answer options: Yes or no |

1. How would you describe your **general health status** after you had COVID-19 compared to before COVID-19?

Answer options: worse, better, or same as before COVID-19?

1. Have you been seen by a doctor because of the long-term effects after COVID-19?

Answer options: Yes or no

1. Have you been given any form of treatment for the long-term effects after COVID-19?

Answer options: Yes or no.

| If yes: |  |  |
| --- | --- | --- |
|  | 26.1 | Please elaborate on which treatment for long-term effects you have received.  A text box appears in which participants can write which other symptoms/long-term effects they have had. |

1. Have you experienced any **other long-term effects** after your COVID-19 that we have not asked about in this questionnaire?

Answer options: Yes or no

| If yes: |  |  |
| --- | --- | --- |
|  | 27.1 | Please elaborate on which **other long-term effects** you have experienced.  A text box appears in which participants can write which other symptoms/long-term effects they have had. |

1. Do you have any further comments?

Answer options: Yes or no

| If yes: |  |  |
| --- | --- | --- |
|  | 28.1 | A text box appears in which participants can elaborate on any further comments. |
