# Supplementary material for: Post-COVID-19 condition after SARS-CoV-2 infection during pregnancy: a population-based questionnaire cohort study
Source: Front Med (Lausanne). 2026 Jan 21;12:1674554. doi: 10.3389/fmed.2025.1674554 (PMC12868251; doi:10.3389/fmed.2025.1674554)
Supplement: Supplementary file 2 [file Table_2.docx]

| **Supplemental file S2.** Association between post-COVID-19 condition and trimester of the primary SARS-CoV-2 infection, admission due to SARS-CoV-2 infection due to symptoms of COVID-19, and whether the participants had symptoms during the primary infection with SARS-CoV-2. | | | | | |
| --- | --- | --- | --- | --- | --- |
|  |  | Included in analyses  (*n* = 920), *n* | Post-COVID-19 condition, *n* | *p-*value | OR (95% CI) |
| Symptoms of COVID-19 during the primary infection with SARS-CoV-2 | Yes | 773 | 580 (79.5%) | < 0.001 | 4.88 (3.37–7.07) |
|  | No | 147 | 56 (38.1%) |  |  |
| Pregnancy length (trimester) at primary infection with SARS-CoV-2 * | 1^st^ | 131 | 95 (72.5%) | 0.624 | - |
|  | 2^nd^ | 494 | 342 (69.2%) |  |  |
|  | 3^rd^ | 292 | 198 (67.8%) |  |  |
| Admitted due to primary SARS-CoV-2 infection | Yes | 28 | 21 (75.0%) | 0.502 | 1.34 (0.57–3.26) |
|  | No | 892 | 616 (69.1%) |  |  |
| OR = Odds ratio; 95% CI = 95% confidence interval  * missing, *n* = 3 | | | | | |
